# Correlation between 5-fluorouracil metabolism and treatment response in two variants of C26 murine colon carcinoma

**DOI:** 10.1038/sj.bjc.6601162

**Published:** 2003-08-12

**Authors:** Y J L Kamm, G J Peters, W E Hull, C J A Punt, A Heerschap

**Affiliations:** 1 Department of Medical Oncology 550, University Medical Center Nijmegen, PO Box 9101, 6500 HB Nijmegen, The Netherlands; 2 Department of Medical Oncology, VU University Medical Center, PO Box 7057, 1007 MB Amsterdam, The Netherlands; 3 Central Spectroscopy Department, German Cancer Research Center, PO Box 101949, D-69009 Heidelberg, Germany; 4 Department of Radiology, University Medical Center Nijmegen, PO Box 9101, 6500 HB Nijmegen, The Netherlands

**Keywords:** ^19^F magnetic resonance spectroscopy, 5-fluorouracil, C26 murine colon carcinoma

## Abstract

Following an i.p. dose of 150 mg kg^−1^ 5-fluorouracil (5-FU), drug uptake and metabolism over a 2-h period were studied by *in vivo* ^19^F magnetic resonance spectroscopy (MRS) for the murine colon carcinoma lines C26-B (5-FU-insensitive; *n*=11) and C26-10 (5-FU-sensitive; *n*=15) implanted s.c. in Balb/C mice. Time courses for tumour growth, intracellular levels of FdUMP, thymidylate synthase (TS) activity, and 5-FU in RNA were also determined, and the effects of a 9.5-min period of carbogen breathing, starting 1 min before drug administration, on MRS-detected 5-FU metabolism and tumour growth curves were examined. Both tumour variants generated MRS-detectable 5-FU nucleotides and showed similar initial growth inhibition after treatment. However, the growth rate of C26-B tumours returned to normal, while the sensitive C26-10 tumours, which produced larger fluoronucleotide pools, still showed moderate growth inhibition. Carbogen breathing did not significantly influence 5-FU uptake or fluoronucleotide production but did significantly enhance growth inhibition in C26-10 tumours. While both tumour variants exhibited incorporation of 5-FU into RNA and inhibition of TS via FdUMP, clearance of 5-FU from RNA and recovery of TS activity were greater for the insensitive C26-B line, indicating that these processes, in addition to 5-FU uptake and metabolism, may be important determinants of drug sensitivity and treatment response.

Colorectal cancer is the third most common cancer in the Western world. Fluoropyrimidine chemotherapy with agents such as 5-fluorouracil (5-FU) is the main method of treatment, but is effective in only a small percentage of patients. More insight into the pharmacokinetics and intracellular metabolism of 5-FU in tumour tissue is essential for a better understanding of the variability of patient response and for the development of improved therapy protocols.

The efficacy of 5-FU treatment depends primarily on tumour uptake of 5-FU and its intracellular anabolic conversion to cytotoxic fluoronucleotides. ^19^F magnetic resonance spectroscopy (MRS) is a powerful method for assessing these aspects *in vivo* since it allows the noninvasive detection and quantitation of 5-FU and its metabolites in tumour during therapy. Such experiments have been described for a number of animal models (Stevens *et al*, 1984; McSheehy *et al*, 1989; Koutcher *et al*, 1991; Sijens *et al*, 1991). However, the clinically relevant problem of why various tumours and patients exhibit widely differing sensitivities to 5-FU therapy has, up to now, not been investigated in detail by this method.

The present study used ^19^F MRS at 4.23 T to monitor *in vivo* in a mouse model the uptake and metabolism of 5-FU in two variants of the C26 murine colon carcinoma. These variants exhibit different sensitivities to 5-FU as demonstrated by the finding that a standard 5-FU treatment protocol cured all animals bearing the sensitive C26-10 tumour, while the relatively insensitive C26-B tumour showed only a two-fold increase in doubling time (van Laar *et al*, 1996a). In addition to the MRS results, the effects of 5-FU therapy on tumour growth *in vivo* and on biochemical parameters determined in tumour extracts (e.g., incorporation of 5-FU into RNA; concentration and activity of the target enzyme thymidylate synthase [TS]) were also examined. Furthermore, we have studied the effect of a short carbogen breathing period on the efficacy of 5-FU treatment for these two tumour variants since carbogen breathing causes vasodilatation and, therefore, may increase tumour perfusion and enhance delivery of 5-FU (McSheehy *et al*, 1998; Howe *et al*, 1999; Kamm *et al*, 2000).

## MATERIALS AND METHODS

### Chemicals

5-FU was obtained from Teva (Mijdrecht, the Netherlands) as a saline solution (50 mg ml^−1^). Carbogen gas (95% O_2_/5% CO_2_) was obtained from Hoekloos (Schiedam, the Netherlands).

### Tumour model

Female Balb/C mice, 8–12 weeks old, were obtained from the Central Animal Laboratory of our university. Two variants of the C26 murine colon carcinoma were employed: C26-B, relatively insensitive to 5-FU; C26-10, sensitive to 5-FU (van Laar *et al*, 1996a). Tumour tissue fragments (diameter: 3 mm) were implanted s.c. in the right flank of each mouse. The growing tumours protruded from the body surface approximately as a half sphere, and tumour volume (cm^3^) was estimated from three orthogonal diameter measurements (*x*, *y*, *z*) as volume=0.5(*xyz*) (Peters *et al*, 1987). Eleven to 12 days after implantation, C26-B tumour volumes were 0.68±0.49 cm^3^ (mean and s.d., *n*=22) while C26-10 tumour volumes were 0.58 ± 0.29 cm^3^ (*n*=24). Mice were divided into groups based on estimated tumour volume, and stratified randomisation was carried out over the different treatment groups. All experimental procedures in the following sections were approved by the local ethics committee for the use of animals according to the standards required by the UKCCR guidelines (Workman *et al*, 1998).

### Treatment

At days 11–12 after implantation (tumour volumes in the range 0.07–1.77 cm^3^), two groups of tumour-bearing mice (C26-B, *n*=11; C26-10, *n*=15) were prepared for 5-FU therapy and ^19^F MRS as follows. Each mouse was canulated i.p. under inhalation anaesthesia using a mixture of enflurane (1.5%), oxygen (29.5%), and nitrous oxide (69%) and mounted in the magnet bore of the MR spectrometer. Core temperature was monitored with a rectal temperature probe and maintained under anaesthesia at 37°C by means of a regulated warm water blanket. 5-fluorouracil was administered i.p. as a 150 mg kg^−1^ bolus by injection of 0.1 ml g^−1^ body weight of 5-FU (1.5 mg ml^−1^) in standard saline over a period of 20 s.

A second, analogous series of experiments including carbogen breathing was performed with separate groups of mice bearing C26-B (*n*=11) and C26-10 (*n*=9) tumours. The animals were prepared for MRS as above, but 1 min prior to 5-FU administration the nitrous oxide and oxygen components of the anaesthesia were replaced with carbogen (2 l min^−1^). After administration of the 5-FU bolus, carbogen breathing was continued for an additional 8.5 min. The carbogen gas was then replaced by oxygen/nitrous oxide for the remainder of the experiment.

The carbogen breathing time used in this study was chosen on the basis of some known vascular responses. (i) After 1 min of carbogen breathing, the oxyhaemoglobin and deoxyhaemoglobin concentrations in human glioma xenografts were found to be close to their final steady-state values (van der Sanden *et al*, 1999). (ii) Upon carbogen breathing, an increase in tumour *p*O_2_ has been reported for both animals and patients; a maximum was reached in patients after 1–6 min (Guichard *et al*, 1994). (iii) In a clinical study of head and neck tumours, the return to precarbogen levels of oxygenation in tumours occurred within ca. 1 min after the end of carbogen exposure (Martin *et al*, 1993). (iv) In clinical radiotherapeutic studies, carbogen breathing was applied 4 min before and continued during radiotherapy for a total exposure of ca. 15 min (Kaanders *et al*, 1995). Carbogen breathing reached its maximal effect within ca. 10 min.

In our study, the purpose of carbogen breathing was to induce a short vasodilation and possibly improved uptake or trapping of 5-FU in tumour after bolus injection. Therefore, carbogen breathing was initiated 1 min before 5-FU injection and continued for one MR data acquisition block of 8.5 min, for a total exposure of 9.5 min.

### Tumour growth

Tumour volume and body weight were monitored twice weekly for 4 weeks following day 0, the day of 5-FU therapy and MRS. For each animal, the measured tumour volumes were divided by the volume at day 0 to give the normalised volumes used in the subsequent analysis. Antitumour activity was evaluated in terms of the following parameters: AUC, the area under the tumour volume growth curves; TD, the tumour doubling time, estimated as the time required for tumours to double in volume after the beginning of treatment; the growth-delay factor

GDF = (TD_treated_ − TD_control_)/TD_control_(1)

which provided a measure of the increase in doubling time caused by treatment (van Laar *et al*, 1996a). Excluded from the analysis were three mice that underwent histological examination and three mice with less than three observations. In a previous study (van Laar *et al*, 1996a), the average TD for untreated C26-10 and C26-B tumours were 4.1 and 2.9 days, respectively, and these values were used as historical control values TD_control_. Toxic side effects were evaluated as mean weight lost (WL) over the time period day 0 to day 28 (as % of weight on day 0), the last day of the experiment, at which point the mice were killed by cervical dislocation.

### ^19^F MR spectroscopy

^19^F MR spectra were measured at 169.457 MHz using a 4.23-T Oxford vertical-bore magnet (bore diameter=75 mm) and a SMIS electronics console. A home-built, two-turn surface coil with an internal diameter of 13 mm, tuneable to ^1^ H or ^19^F, was placed around the s.c. tumour located in the right flank. The sensitive volume of the coil was centred on the tumour tissue. Magnetic field homogeneity was adjusted by shimming on the tissue H_2_O signal to reach a line width of less than 70 Hz (0.4 ppm).

For ^19^F MRS, a simple pulse-acquire sequence was used with a spectral width of 10 kHz, a time domain of 4 K points, a rectangular radiofrequency pulse of length 20 *μ*s (ca. 70° flip angle at the centre of the surface coil), and a repetition time TR=0.5 s. A flip angle *β*=70° is optimal in repetitive pulsing experiments for TR/*T*_1_=1.07, giving >96% of the maximum possible signal-to-noise ratio. The ^19^F *T*_1_ values for 5-FU and its metabolites have not been measured under our *in vivo* conditions, but are expected to lie in the range of ca. 0.12–1.7 s, measured at 1.5 T (Li *et al*, 1998; Klomp *et al*, 2003), and will depend not only on molecular weight, but also to some extent on binding equilibria with proteins and local concentrations of paramagnetic metal cations, for example. Thus, the detected ^19^F signal intensity for a given small tissue voxel will be proportional to

*C*_met_*B*_1_*F*_sat_sin*β*(2)

where *C*_met_ is the local metabolite concentration.

*F*_sat_ = (1 − E1)/(1 − E1 cos*β*), with E1 = exp(−TR/T_1_)(3)

is the local saturation factor (depending on TR/*T*_1_ and the position-dependent flip angle), and *B*_1_ (the local rf field strength produced by the coil) is proportional to the local detection sensitivity. The total signal will be the sum over all detectable voxel signals and will depend on tumour size, metabolite concentration distribution, as well as the spatial dependence of the saturation and sensitivity factors. For a 70° flip angle, saturation factors (signal intensity relative to the case TR≫*T*_1_) will be 0.72–1.0 for TR/*T*_1_=1 (expected to apply for fluoronucleotides) or could be as low as 0.35 for the extreme condition TR/*T*_1_=0.3 (corresponding to *T*_1_=1.7 s in our experiments).

Thus, in any given ^19^F spectrum, the relative signal areas for 5-FU and its various metabolites will depend on the saturation factors and may only crudely reflect their mean relative tissue concentrations. However, we were interested in changes in signal areas over time for a given metabolite and tumour (time course), and signal *vs* time for a given metabolite should be roughly proportional to an effective mean tissue concentration *vs* time if saturation and sensitivity factors and the *distribution* of the metabolite relative to the surface coil do not change dramatically.

^1^ H decoupling was not used since the *in vivo* linewidths were much larger than ^3^*J*_HF_ (5 Hz) for the fluorouracil moiety. The time-domain ^19^F MR data were stored as sequential 8.5-min blocks (1024 transients per free induction decay (FID)), beginning at the moment of 5-FU injection and continuing over a period of 2 h.

To check the localisation of the MR-sensitive volume, we used the same experimental set-up to obtain ^1^ H images and estimated that nearby liver tissue represented less than 5% of the detected volume. The absence of a significant contribution from liver or the intraperitoneal volume to the ^19^F MR signals was further confirmed by two ^19^F MRS measurements performed on animals *without* tumour but treated as described above. In these cases, very little signal from 5-FU or its metabolites was detected.

^1^ H MR spectra were recorded at 180.130 MHz before and after ^19^F MR acquisition with the rf coil still tuned to ^19^F (rf pulse length 20 *μ*s, rf power adjusted to give a 70° flip angle at the centre of the coil, 64 transients, repetition time TR=1 s). These conditions are similar to those used for ^19^F (optimal S/N for TR/*T*_1_=1.07). The resulting signal from tissue water will represent a complex sum of product terms for each voxel, as described above for the ^19^F measurements. Since the flip angle and TR/*T*_1_ parameters are similar for the ^19^F and ^1^ H measurements, the tissue volumes ‘seen’ by the two experiments should be similar (^1^ H *T*_1_ were not measured but are expected to be in the range 0.5–2.5 s, leading to saturation factors in the range 0.33–1.0 for a 70° flip). Assuming tissue water concentration is roughly constant, then the ^1^ H signal intensity should increase with tissue volume (tumour size) in the same manner as the ^19^F signal will increase with tumour size if the detected metabolite has a relatively uniform distribution with constant mean concentration. Therefore, the ^1^ H signal integral was used to normalise the ^19^F signal integrals obtained from different tumours and to provide a crude form of compensation for differences in signal intensity caused by differences in tumour volume alone. The ^1^ H MR spectra were recorded before and 2 h after carbogen breathing to avoid effects of carbogen on the amplitude or integral of the water ^1^ H signal (relaxation effects due to change in paramagnetic oxygen concentration).

### Quantitative analysis of ^19^F MR spectra

**Figure 1:**
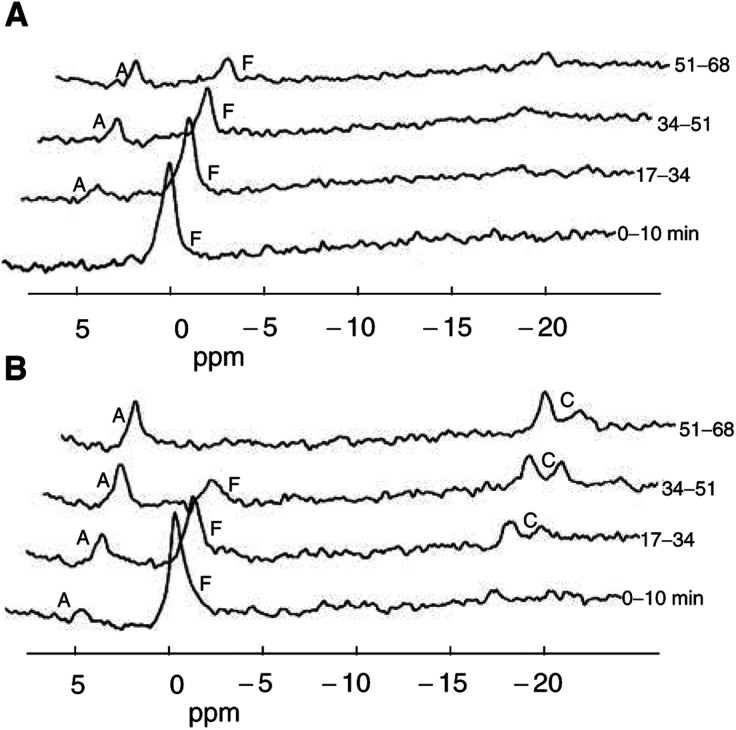
Sequential ^19^F MR spectra (4.23 T) obtained *in vivo* from a subcutaneous C26-B ((**A**) 0.39 cm^3^) or C26-10 ((**B**) 0.45 cm^3^) murine colon carcinoma in a Balb/C mouse following injection of a bolus i.p. dose of 5-FU (150 mg kg^−1^) at time *t*=0. Data acquisition was started simultaneously with the 5-FU injection, and sequential 8.5-min data blocks (FIDs) were stored. To improve the S/N for the presentation, each spectrum shown was obtained by adding two successive 8.5-min acquisitions (equivalent to 17 min of time averaging) before Fourier transformation. Peak labels: F=unmetabolized 5-FU (chemical shift defined as 0 ppm); C=the major catabolites FUPA (−16.5 ppm) and FBAL (−19.2 ppm); A=a composite signal at ca. 5 ppm representing the overlapping peaks from all fluoronucleotides in oxy (FUMP, FUDP, FUTP, FUDP-hexoses) and deoxy (FdUMP) forms.

For the presentation in Figure 1

, two blocks of ^19^F time-domain data (17-min acquisitions) were added and multiplied by an exponential window function (line broadening of 20 Hz) before Fourier transformation to the frequency domain. The ^19^F MR resonance frequency observed for 5-FU *in vivo* was defined as the reference point for all chemical shift measurements (0.0 ppm, equivalent to ca. −93.5 ppm relative to trifluoroacetate). Quantitative analysis of the ^19^F and ^1^ H MR spectra was performed by fitting the time-domain signal using the software package MRUI (van den Boogaart *et al*, 1995) and assuming Lorentzian line shapes.

Relative tissue levels (in arbitrary units) for 5-FU (F), total detectable anabolites (A, free fluoronucleotides not bound to or incorporated in macromolecules), and total catabolites (C) were estimated from the signal integrals for 5-FU, the spectral region 4.2–5.2 ppm corresponding to FUMP, FdUMP, FUTP, etc., and the region −16 to −20 ppm corresponding to the major catabolites *α*-fluoro-*β*-ureidopropionic acid (FUPA: −16.5 ppm) and *α*-fluoro-*β*-alanine (FBAL: −19.2 ppm). All ^19^F signal integrals were normalised by dividing by the mean value of the H_2_O signal integral obtained from the two ^1^ H reference spectra. The normalised ^19^F MR signal integrals were then considered to represent mean relative metabolite concentrations per unit volume of tissue (averaged over the sensitive volume detected by the surface coil) and, for a given metabolite, can be compared across all animals and groups for all tumour volumes. This technique assumes that the water ^1^ H signal and the metabolite ^19^F signals exhibit the same dependence for intensity *vs* tumour volume, which should be a reasonable assumption if both tissue water and 5-FU metabolites have similar distributions, as discussed above. No corrections were attempted for possible differences in ^19^F *T*_1_ and saturation factors for the various metabolites, which may influence their relative signal integrals for a given tumour spectrum. The tissue half-life (*t*_1/2_) of 5-FU was estimated from the ^19^F MRS time course as the time required for 5-FU to decrease from its maximum concentration to half that value.

### *Ex vivo* determination of 5-FU, FdUMP, and 5-FU incorporation into RNA

Two groups of mice bearing C26-B or C26-10 tumours received 5-FU treatment (150 mg kg^−1^ bolus, i.p.) as described above for the MRS and growth studies. Tumours were removed at various time points after treatment and immediately frozen in liquid nitrogen. Frozen tumours were pulverised (Peters *et al*, 1986), suspended in three volumes of Tris-HCl buffer (pH 7.4, cooled on ice), and immediately extracted with trichloroacetic acid (final concentration 5%). The supernatant was neutralised and stored until analysis for FdUMP and 5-FU. The acid-insoluble precipitate was used for the isolation of RNA and the measurement of 5-FU incorporation into RNA.

FdUMP concentrations were measured by means of a dilution assay based on the capacity of FdUMP in the tumour extract to inhibit the binding of [6-^3^ H]FdUMP to thymidylate synthase from *Lactobacillus casei* (van der Wilt *et al*, 1992). 5-Fluorouracil was derivatised with penta-fluoro-benzylbromide (Peters *et al*, 2003) and measured with gas chromatography coupled to mass spectrometry (GC–MS) using [^15^N_2_]5-FU as an internal standard, as described previously (Peters *et al*, 1993).

Incorporation of 5-FU into RNA was determined using the RNA isolated from the first precipitation step. Incubation with RNAse (RNA breakdown), alkaline phosphatase (dephosphorylation), and uridine phosphorylase (breakdown of fluorouridine to 5-FU) resulted in the stepwise degradation of RNA to give 5-fluoro-uridine-3′-monophosphate (3′-FUMP), 5-FUrd, and finally 5-FU, which was analysed by GC–MS (Peters *et al*, 1995).

### Evaluation of TS inhibition

Two assays were used to evaluate TS levels in the tumour extracts described above. The ligand-binding assay using [6-^3^ H]FdUMP gave a measure of the number of free FdUMP binding sites available on TS after 5-FU treatment (van der Wilt *et al*, 1992). The TS catalytic activity assay measured the rate of conversion of [5-^3^ H]dUMP into dTMP and ^3^H_2_O (van Laar *et al*, 1996b). Tumour volumes for treated and untreated control mice were in the range 0.20–0.30 cm^3^. These tumours were generally chosen to be smaller than those used for ^19^F MRS (where larger tumours provide more MR signal) in order to avoid the development of an unacceptably large tumour burden during the course of the experiment when treatment was ineffective. However, in the volume range tested, no significant differences in TS levels were found, as long as the tumours were in the exponential growth phase (van der Wilt *et al*, 1992; van Laar *et al*, 1996a, 1996b).

### Statistics

Statistical analyses were performed using the SAS software package (SAS Institute, Cary, NC, USA). The parameters *C*_max_ and AUC in Table 1

**Table 1:** Median parameter values for 5-FU metabolism determined by *in vivo*^19^F MRS of tumour-bearing mice

		***C*max^b^**		**AUC^c^**	
		**Median (min, max)**		**Median (min, max)**	
**Metab.**	**Tumour**	**5-FU**	**5-FU+carbogen**	**5-FU**	**5-FU+carbogen**
5-FU	C26-B	0.98 (0.44, 3.01)	0.78 (0.51, 1.61)	39 (15, 72)	34 (13, 77)
	C26-10	0.78 (0.38, 2.95)	1 (0.45, 2.07)	27 (11, 80)	38 (17, 85)
		*P*=0.82		*P*=0.63	
					
Anab.	C26-B	0.35 (0, 0.59)	0.36 (0.21, 0.50)	20 (0, 34)	25 (9, 35)
	C26-10	0.65 (0.31, 2.81)	0.59 (0.45, 2.07)	50 (20, 190)	48 (33, 139)
		*P*=0.0004		*P*=0.0001	
					
Catab.	C26-B	0.51 (0, 3.58)	0.36 (0, 0.76)	17 (0, 129)	14 (0, 31)
	C26-10	0.35 (0, 3.56)	0.32 (0, 0.57)	13 (0, 96)	11 (0, 24)
		*P*=0.23		*P*=0.36	
					
		**5-FU *t*_*1/2*_ (min)^d^**			
	C26-B	25.5 (17, 51)	25.5 (8.5, 51)		
	C26-10	25.5 (8.5, 42.5)	34 (25.5, 34)		
		*P*=0.51			

a Balb-C mice bearing murine colon carcinoma (s.c.) were examined by ^19^F MRS following a bolus injection of 150 mg kg^−1^ 5-FU (C26-B, *n*=11; C26-10, *n*=15) or following 5-FU treatment combined with 9.5 min carbogen breathing (C26-B, *n*=11; C26-10; *n*=9). Pharmacokinetic parameters were evaluated for 5-FU, the total NMR-detectable anabolite pool, and the sum of the catabolites FUPA+FBAL. Two-way ANOVA was performed with transformed data (skewed distributions, see Materials and Methods) and gave the *P* values shown for the main effect of tumour type; for the main effect of carbogen, *P*>0.05 was found in all cases.

b *C*_max_=mean of max tissue concentration in arbitrary relative units, determined from ^19^F signal integrals normalised to constant tissue volume using the ^1^ H signal from tissue water.

c AUC=area under the concentration–time curve (from 0 to 119 min), normalised to constant tissue volume.

d *t*_1/2_=half-life of 5-FU (time for decay from *C*_max_ to 50% of *C*_max_).

have highly skewed distributions; therefore, median, min, and max values are presented. For the two-way ANOVA comparisons, which require distributions close to normality, skewness was reduced by a *log* transformation of the *C*_max_ data and a *square-root* transformation of AUC. For Table 2

**Table 2:** 5-FU pharmacokinetic parameters and inhibition of TS determined from extracts of C26-B and C26-10 tumours

	**Time C26-B**			**C26-10**		
**Parameter**		**Mean±s.d.**	***n***	**mean±s.d.**		***P***<
FdUMP	2 h	138±30	6	179±42	5	
(pmol g^−1^ wet wt.)	4 h	107±22	6	147±78	5	
	24 h	22.0±2.7	4	32±15	4	
	48 h	13.3±1.8	4	46±35	4	
						
5-FU	7 days	0.14±0.03	4	0.82±0.34	4	0.05
(nmol g^−1^ wet wt.)	10 days	0.12±0.02	4	0.79±0.22	4	0.02
						
5-FU-RNA	2 days	7.41±0.30	4	2.25±1.55	4	0.01
(pmol 5-FU *μ*g^−1^ RNA)	3 days	6.63±0.20	4	1.71±0.53	4	0.001
	7 days	0.25±0.025	4	0.40±0.01	4	0.001
	10 days	0.06±0.01	4	0.16±0.01	4	0.001
						
TS catalytic activity	*Controls*	5.73±0.41	8	2.85±0.20	8	0.001
(nmol h^−1^ mg^−1^ protein)	1 day	0.72±0.10	4	1.09±0.03	4	0.001
	3 days	1.21±0.18	4	1.72±0.48	4	
	7 days	9.96±0.15	4	5.73±0.43	4	0.001
	10 days	8.55±1.24	4	1.92±0.28	4	0.001
						
FdUMP binding	*Controls*	122±33	8	93±12	8	
(fmol g^−1^ wet wt.)	1 day	32.5±2.3	4	20.3±1.5	4	0.001
	3 days	31.5±5.4	4	22.6±3.8	4	0.05
	7 days	201±44	4	165±33	4	
	10 days	190±31	4	145±23	4	

a Tumours were excised at the times shown in column 2, following 5-FU treatment (day 0: bolus i.p., 150 mg kg^−1^) or were taken from untreated controls with tumours of similar size. After excision, tumours were immediately frozen in liquid nitrogen. The *P* values <0.05 are given for comparisons between the two tumour types (two-sided *t*-tests, assuming equal variance).

differences, between mean values of parameters for different groups were analysed by the unpaired Student's *t*-test (two-sided, equal variance). The two-sample Wilcoxon rank-sum test was used to evaluate differences in *t*_1/2_ for 5-FU (Table 1) and the tumour doubling time (TD, Table 3

**Table 3:** Response of C26-B and C26-10 tumours to 5-FU treatment with or without carbogen breathing

**Tumour**	**(Group) Treatment^a^**	***n***	**TD^b^ (days)**	**GDF^c^**	**WL (%)^d^**	***n***
C26-B	*Controls*		2.9±1.1			
	(1) 5-FU	9	10.2 (6.9, 17.1)	2.52	5.6±8.4	10
	(2) 5-FU+carbogen	11	10.2 (6.9, 19.6)	2.52	11.1±5.7	11
						
C26-10	*Controls*		4.1±2.6			
	(3) 5-FU	11	13.3 (9.3, >28)	2.23	11.9±8.3	13
	(4) 5-FU+carbogen	10	>28 (9, >28)	>5.83	11.1±3.3	10

a The Group number corresponds to the labelling in Figure 2.

b Tumour doubling time is presented as mean±s.d. for untreated historical controls or as median (min, max) measured from treatment day 0 (excluding mice with<three observations). The symbol>indicates that doubling did not occur before the end of the experiment on day 28. One C26-10 tumour treated with 5-FU and one C26-10 tumour treated with 5-FU+carbogen showed complete remission. Significance of comparisons (Wilcoxon rank-sum test): (1) *vs* (3) *P*=0.048; (2) *vs* (4) *P*=0.002; (1) *vs* (2) *P*=0.62; (3) *vs* (4) *p*=0.033.

c Growth delay factor (see Materials and Methods).

d Mean weight loss per time point (see Materials and Methods) (mean±s.d., including mice with<three observations).

). Tumour growth curves following treatment were analysed by a distribution-free analysis of variance for repeated measurements (Koziol *et al*, 1981).

## RESULTS

### ^19^F MRS of C26-B and C26-10 tumours following 5-FU treatment

In Figure 1, representative time-course spectra (17-min time averages) are shown for the two tumour types without carbogen breathing. Up to four ^19^F MR signals were observed. The signal F at 0 ppm on the chemical shift scale represents unmetabolised 5-FU; it reached a maximum intensity within the interval 0–17 min after drug administration and decreased thereafter. The cytotoxic anabolite pool derived from 5-FU (all fluoronucleotides such as FdUMP, FUTP, and FUDP-hexoses) gave one composite peak (A) at ca. 4.8 ppm, which could be detected in the second or third measurement block (8.5–25.5 min) and increased to a maximum plateau level after ca. 60–80 min when 5-FU was no longer detectable. Higher levels of anabolites (A) were generally observed for the 5-FU-sensitive C26-10 tumours (Figure 1B). Catabolism of 5-FU (primarily in the liver) results initially in 5,6-dihydro-5-fluorouracil (DHFU: not detected at −33.0 ppm), which is rapidly converted to the intermediate FUPA (−16.5 ppm) and finally to FBAL (−19.2 ppm). These latter two catabolites appear to the left and right of the label C in Figure 1.

The quantitative results from the MR experiments were determined by analysis of the original data with 8.5-min time resolution and are summarised in Table 1. For the fluoronucleotide anabolites, both the maximum achieved concentration *C*_max_ as well as the AUC were significantly higher for the 5-FU-sensitive C26-10 tumour compared to C26-B (*P*<0.001), with or without carbogen breathing. In contrast, *C*_max_ and AUC for catabolites and 5-FU as well as the half-life *t*_1/2_ of 5-FU were not significantly different for the two tumour types (*P*>0.2). Treatment with carbogen had no effect on the mean values for the parameters of 5-FU metabolism for either tumour variant (*P*>0.05 in all cases), but resulted in a significant reduction in the range of *C*_max_ and AUC for catabolites.

### Time course of metabolite concentrations and TS activity as determined in tumour tissue extracts

Separate groups of tumour-bearing mice were given the same 5-FU treatment as for the MRS studies (day 0=11–12 days after implantation), and tumours were excised at various times thereafter. Extracts of the excised tumour tissues were used to determine the time courses for metabolites and TS enzyme activity presented in Table 2. Although the mean FdUMP concentrations over 2–48 h were somewhat higher in the sensitive C26-10 tumours compared to the insensitive C26-B, these differences were not statistically significant (*P*>0.05). On the other hand, the initial incorporation of 5-FU into RNA at day 2 or 3 after treatment was about a factor of 3–4 lower in C26-10 compared to C26-B (*P*<0.001 on day 3). However, at day 7 or 10 there was a more rapid decrease in FU-RNA in the insensitive C26-B tumours to values less than those observed for C26-10, and residual FU levels in C26-B were about one-sixth the values for C26-10.

Thymidylate synthase catalytic activity in untreated C26-10 control tumours was found to be about half the level in C26-B controls (*P*<0.001). For both tumours, TS activity decreased significantly 1 day after 5-FU administration, rebounded to about twice the control level by day 7, and finally decreased towards control levels on day 10. The relative inhibition of TS (compared to controls) at day 1 was significantly higher for C26-B (factor 8) *vs* C26-10 (factor 2.6), while the relative degree of overshoot at day 7 was similar.

In untreated controls, the mean number of FdUMP binding sites in the sensitive C26-10 tumours was 76% of the mean for C26-B (difference not statistically significant). Following 5-FU treatment, the number of binding sites for both tumour types decreased by about a factor of 4 at day 1 and rebounded with the same overshoot behaviour at day 7 as observed for TS activity. The number of binding sites for C26-10 relative to C26-B remained at 60–80% over the 10-day time course.

### Tumour histology

Histological examinations of six individual untreated tumours of C26-B and of C26-10 murine colon carcinoma were performed. Both variants were poorly differentiated adenocarcinoma with less than 15% necrosis on days 11–12 after implantation, and no clear differences in histology or vascularisation were observed.

### Effect of 5-FU treatment on tumour growth

Normalised growth curves for s.c. C26-B and C26-10 tumours in Balb/C mice following i.p. bolus 5-FU treatment are shown in Figure 2

**Figure 2:**
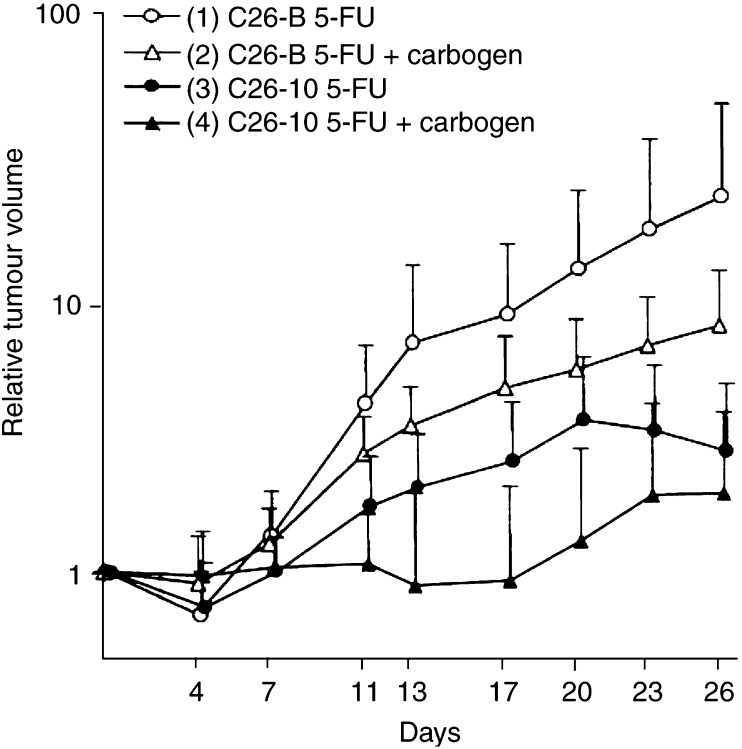
Normalised growth curves (relative tumour volume: mean±s.d.) for s.c. implanted colon carcinoma C26-B and C26-10 in Balb/C mice after i.p. bolus treatment with 150 mg kg^−1^ 5-FU alone (C26-B, *n*=9; C26-10, *n*=11) or in combination with 9.5-min carbogen breathing (C26-B, *n*=11; C26-10, *n*=10). Two-way ANOVA indicates that differences between tumour variants are highly significant with or without carbogen: group 1 *vs* 3, *P*=0.02; group 2 *vs* 4, *P*=0.003. A significant effect of carbogen was observed only for the more slowly growing, 5-FU-sensitive C26-10 tumours: group 1 *vs* 2, *P*=0.35; group 3 *vs* 4, *P*=0.03.

, and the parameters doubling time (TD), growth delay factor (GDF), and weight loss (WL) derived from these data are shown in Table 3. Note that the two tumour variants have inherently different growth rates in untreated controls, with the less sensitive C26-B tumour showing faster growth (TD=2.9 days) compared to the more sensitive C26-10 tumour (TD=4.1 days).

5-Fluorouracil treatment without carbogen led to moderate regression at day 4 for both tumour types, but growth continued again after day 7 with C26-B exhibiting the higher growth rate. Owing to 5-FU, the doubling times calculated from day 0 increased to 10.2 days (C26-B, group 1 in Figure 2) and 13.3 days (C26-10, group 3) and were significantly different (*P*=0.048), but the growth delay factors were similar (2.52 *vs* 2.23).

When carbogen was applied during treatment, there was no significant change in the initial response of C26-B tumours (group 2: TD, GDF unchanged), but there was a moderate decrease in the mean growth rate after day 11 for the carbogen group. A significant effect of carbogen was observed, however, for the 5-FU-sensitive C26-10 tumours (group 4) with the median doubling time increasing to >28 days (*P*=0.033 *vs* group 3, *P*=0.002 *vs* group 2), giving a GDF>5.8. Tumour regrowth began only after an average of 17 days. Following the single bolus 5-FU treatment, complete tumour remission was observed only for two out of 26 C26-10 tumours (one with and one without carbogen).

The mean percentage weight loss (WL) over the time period days 0–28 was 6% for the C26-B tumour-bearing animals treated with 5-FU alone and 12% in the other three groups. These values were considered to be within acceptable limits.

## DISCUSSION

Here, we present the first ^19^F MRS results (0–2 h time course) for two variants of the C26 tumour line with different sensitivities to 5-FU, along with biochemical data derived from tissue extracts (time courses from 2 h postdrug to up to 10 days). Our ^19^F MRS results show that the more sensitive C26-10 tumour exhibits a significantly higher conversion of 5-FU to fluoronucleotides (cytotoxic anabolites) compared to the insensitive, more rapidly growing C26-B tumour (*C*_max_ and AUC in Table 1). These results support previous findings that positive therapy response correlates with the concentration of anabolites achieved in tumour tissue, either directly or with the aid of modulators (McSheehy *et al*, 1989, 1992; Koutcher *et al*, 1991; Sijens and Ng, 1992; Findlay *et al*, 1993; Tausch-Treml *et al*, 1996; Holland *et al*, 1997). The broad anabolite peak observed in Figure 1 represents the complete family of fluoronucleotides (mono-, di-, triphosphates; oxy- and deoxy forms) which cannot be further distinguished with *in vivo* ^19^F MRS. Previous studies have shown that the major contributors to the anabolite peak are probably FUMP, FUDP, FUTP, and FUDP-hexoses (Lutz and Hull, 1999). The ratio FUTP/FdUMP may be as high as 50 (Benz and Cadman, 1981; Peters *et al*, 1986), and the concentrations of free FdUMP and its ternary complex with TS are generally <1 *μ*M (Washtien, 1984). Thus, these key species cannot be detected by *in vivo* ^19^F MRS. However, the concentration of FdUMP in C26 tumours (van der Wilt *et al*, 1992; van Laar *et al*, 1996b) and in patients (Peters *et al*, 1993) has been determined by other methods and was found to be high enough to inhibit TS effectively.

Table 1 shows that there are no significant differences between the tumour variants for uptake of 5-FU (*C*_max_, AUC) or its half-life (*t*_1/2_). Therefore, these parameters cannot account for the higher anabolite levels observed in C26-10 compared to C26-B. Differences in catabolism of 5-FU in tumour via dihydropyrimidine dehydrogenase (DPD) can also be ruled out since DPD was not detectable in either tumour variant (Visser *et al*, 1996). Thus, 5-FU catabolism in C26 tumours is expected to be insignificant, and the catabolites detected in the spectra of tumour tissue (Figure 1) are considered to result from uptake from the circulation due to the high plasma levels generated by the extremely efficient catabolism (detoxification) of 5-FU in the liver (Grem, 1990). A correlation between 5-FU catabolites in tumour and levels in plasma and surrounding tissue (Naser-Hijazi *et al*, 1991) or levels in kidney (Lutz *et al*, 1991) has been demonstrated in other studies. Since catabolite levels in liver can reach several millimolars following bolus treatment (5–10 times the levels in tumour), even a 5% contribution of liver tissue to the sensitive volume detected by a surface coil can be responsible, at least in part, for the catabolite signals detected.

Thus, enhanced anabolic conversion of 5-FU is likely to be responsible for the higher levels of fluoronucleotides detected by MRS in C26-10 *vs* C26-B tumours. Higher activity of uridine phosphorylase or uridine kinase in C26-10 could result in more rapid formation of FUMP and subsequent conversion to FUTP, FdUMP, and FdUTP, which are responsible for the so-called RNA- and DNA-directed mechanisms of 5-FU cytotoxicity. Both mechanisms may indeed be active, but their relative importance may depend on cell phenotype, cell cycle, and other parameters. Note that despite the higher anabolite levels observed by MRS in C26-10 tumours, the average degree of growth inhibition or regression at day 4 was essentially the same for both tumour types (Figure 2).

Since MRS provides little information on the details of 5-FU cytotoxicity, the biochemical data of Table 2 are of interest. The incorporation of FUTP into RNA (structural damage, misreading of nucleotides) was actually *higher* in the insensitive C26-B tumours at days 2 and 3 after therapy. On the other hand, a more rapid clearance of fluorine from RNA by day 7 was observed for C26-B (faster growth rate, higher RNA turnover) compared to C26-10, and both 5-FU-RNA and free 5-FU levels were lower in C26-B at days 7 and 10. This ability of C26-B tumour cells to clear more rapidly 5-FU from RNA may contribute to the insensitivity of this cell line to 5-FU therapy and the return to a normal growth rate after day 7 (TD=2.7, Figure 2). In contrast, C26-10 tumours showed less initial incorporation of 5-FU into RNA but slower clearance and higher long-term 5-FU levels (days 7 and 10), and these effects are associated with only a partial recovery in growth after day 7 (TD=ca. 6, Figure 2). In spite of this evidence, the relative importance of RNA-directed cytotoxicity in determining therapy response in C26 tumours cannot be precisely defined at this time.

Thymidylate synthase represents a potential target for the DNA-directed mechanism of fluoropyrimidine chemotherapy (Danenberg, 1977). FdUMP produced by intracellular metabolism of 5-FU in tumours is an analogue of dUMP and blocks *de novo* dTMP production by forming a stable covalent ternary complex with TS and a tetrahydrofolate cofactor, leading to a depletion of dTTP needed for DNA synthesis. The relatively insensitive C26-B tumours exhibit a higher growth rate (shorter doubling time) and higher basal TS catalytic activity (Table 2, controls) compared to the 5-FU-sensitive C26-10 tumours (van Laar *et al*, 1996a). In addition, the pools of reduced folates are smaller in C26-B and could limit the achievable degree of TS inhibition (van der Wilt *et al*, 2001). We found that following 5-FU treatment, C26-B tumours generated somewhat lower levels of FdUMP compared to C26-10 (not statistically significant), but for both tumours the concentrations of FdUMP after 2 h were about 1000-fold higher than the number of FdUMP binding sites found in controls. Both types of tumour exhibited a significant reduction in available binding sites and inhibition of TS at days 1 and 3 following 5-FU treatment, and the degree of inhibition was in fact higher for C26-B compared to C26-10. Both tumours exhibited a rebound in FdUMP binding sites and TS activity at days 7 and 10 to levels even higher than controls, indicating induced synthesis of TS. However, the rebound in TS activity in C26-B was larger and longer lasting, analogous to the more efficient clearance of 5-FU from RNA (see above).

Translational regulation of key enzymes has been implicated as a possible mechanism for the development of drug resistance (reviewed by Schmitz *et al*, 2001), and it has been shown that overexpression of TS in breast and colon carcinoma cell lines results in reduced sensitivity to 5-FU treatment *in vitro* (Chu *et al*, 1991a). It has been shown that TS controls (suppresses) its own synthesis (autoregulation) by binding to an untranslated region of TS mRNA, and that FdUMP leads to an induction of TS by deregulation (derepression) of TS synthesis (Chu *et al*, 1991b). It was proposed that this ligand-mediated induction of TS occurs because the ternary complex TS-FdUMP-tetrahydrofolate exhibits weaker binding to TS mRNA and is, therefore, inefficient in suppressing TS synthesis. More recently, Kitchens *et al* (1999) have provided experimental evidence with colon tumour cell lines that the autoregulation model may not be the universal explanation for ligand-mediated induction of TS. In particular, a three-base modification of TS mRNA which abolished TS binding did not interfere with drug-mediated TS induction. It was shown that ligand binding increased the stability of TS towards proteolytical degradation and increased the intracellular half-life of TS several fold, that is by an amount consistent with the observed induction. Thus, it was reasoned that enhanced TS concentrations were not a result of enhanced protein synthesis, but rather decreased degradation due to the stability of the inhibitory ternary complex. Since the complex sequesters FdUMP, it may serve to reduce intracellular levels of FdUMP and sensitivity to 5-FU (Kitchens *et al*, 1999).

Both the translation and stability mechanisms discussed above may, in fact, operate in the cell lines investigated in our study (Peters *et al*, 2002). The information at hand (rebound in TS catalytic activity and available FdUMP binding sites above control levels) does provide evidence that is more consistent with enhanced TS synthesis rather than the stability model. In any event, all of the results presented here suggest that the lower sensitivity of C26-B to 5-FU treatment may not only be due to the lower levels of MRS-detectable fluoronucleotides but perhaps more importantly due to the ability of this cell line to recover more effectively from both RNA- and DNA-directed cytotoxicity via more efficient clearance of 5-FU from RNA and greater TS induction, respectively.

As to the effect of carbogen on 5-FU treatment, we previously found increased levels of 5-FU and its metabolites as a result of carbogen breathing in a different murine colon tumour model (Kamm *et al*, 2000). However, in the current study, carbogen breathing for 9.5 min during 5-FU administration did not significantly affect the MRS-detectable aspects of 5-FU metabolism in either C26 tumour variant. In terms of tumour growth, there was no effect on TD or GDF for C26-B tumours and only a minor decrease in the regrowth rate (TD=3.5 from day 7, Figure 2). However, a dramatic effect of carbogen was observed for the sensitive C26-10 tumours: growth inhibition out to day 17 (Figure 2) with a significant increase in the parameters TD and GDF (Table 3). Since carbogen did not increase the MRS-detected levels of 5-FU or fluoronucleotides at 2 h postdrug administration in the C26 tumours studied, the reason for the prolonged growth delay with C26-10 remains unclear at this time. Certainly, it will be of interest to investigate the biochemical parameters of Table 2 as a function of carbogen exposure.

We note that it has been demonstrated for a rat tumour model (Lemaire *et al*, 1998) that good energy status (NTP/P_i_ ratio) prior to therapy (presumably improved by carbogen) and a high level of fluoronucleotides produced following a 5-FU bolus were associated with tumour regression. However, we do not have appropriate ^31^P-MRS data to address this point for C26. It is known that the effect of carbogen may depend on tumour characteristics such as type, size, vascularisation, perfusion, and pH (Rodrigues *et al*, 1997), but we do not yet have detailed information concerning oxygenation or pH status for the C26 tumours. Furthermore, it may be necessary to extend the carbogen breathing period to account for the resorption delay associated with i.p. *vs* i.v. drug administration (Griffiths *et al*, 2001).

In conclusion, we provide quantitative evidence for differences between two variants of the C26 murine colon carcinoma in terms of *in vivo* 5-FU uptake and anabolic conversion as well as recovery from treatment via clearance of 5-FU from RNA and induction of TS. These differences are consistent with and probably responsible for the observed differences in sensitivity to 5-FU treatment. These findings concerning possible mechanisms or sources of drug resistance may be of immediate clinical relevance since cancer patients exhibit a wide range of sensitivity to fluoropyrimidine therapy.
